# Gene expression correlated with delay in shell formation in larval Pacific oysters (*Crassostrea gigas*) exposed to experimental ocean acidification provides insights into shell formation mechanisms

**DOI:** 10.1186/s12864-018-4519-y

**Published:** 2018-02-22

**Authors:** Pierre De Wit, Evan Durland, Alexander Ventura, Chris J. Langdon

**Affiliations:** 1 Department of Marine Sciences, University of Gothenburg, Strömstad, Sweden; 2 Department of Fisheries and Wildlife and Coastal Oregon Marine Experiment Station, Oregon State University, Newport, Oregon USA; 3 Department of Biological and Environmental Sciences, University of Gothenburg, Fiskebäckskil, Sweden

**Keywords:** *Crassostrea gigas*, Gene expression, Larvae, Ocean acidification, Aragonite, Calcification

## Abstract

**Background:**

Despite recent work to characterize gene expression changes associated with larval development in oysters, the mechanism by which the larval shell is first formed is still largely unknown. In Crassostrea gigas, this shell forms within the first 24 h post fertilization, and it has been demonstrated that changes in water chemistry can cause delays in shell formation, shell deformations and higher mortality rates. In this study, we use the delay in shell formation associated with exposure to CO_2_-acidified seawater to identify genes correlated with initial shell deposition.

**Results:**

By fitting linear models to gene expression data in ambient and low aragonite saturation treatments, we are able to isolate 37 annotated genes correlated with initial larval shell formation, which can be categorized into 1) ion transporters, 2) shell matrix proteins and 3) protease inhibitors. Clustering of the gene expression data into co-expression networks further supports the result of the linear models, and also implies an important role of dynein motor proteins as transporters of cellular components during the initial shell formation process.

**Conclusions:**

Using an RNA-Seq approach with high temporal resolution allows us to identify a conceptual model for how oyster larval calcification is initiated. This work provides a foundation for further studies on how genetic variation in these identified genes could affect fitness of oyster populations subjected to future environmental changes, such as ocean acidification.

**Electronic supplementary material:**

The online version of this article (10.1186/s12864-018-4519-y) contains supplementary material, which is available to authorized users.

## Background

Calcium carbonate (CaCO_3_) particles have been reported to be initially formed in the intracellular compartments of specialized cells that transport the mineral to sites of shell formation in adult molluscs [1] and possibly in larvae. The composition and structure of a protein matrix that is also thought to be deposited by specialized cells [1], determines how CaCO_3_ crystals become organized, forming different isoforms with distinct chemical and physical properties [2]. The most common of these isoforms in marine molluscs are calcite and aragonite. Shells of adult oysters are primarily composed of calcite [3], but the larval oyster shell is composed of aragonite [4].

Aragonite of Pacific oyster larval shells starts to form after 14–18 h post-fertilization under standard culture conditions [5]. Embryos initially exist as unprotected trochophore larvae whereupon a shell gland forms and produces an organic pellicle or periostracum [6], allowing shell deposition to begin between the periostracum and the larval epithelium of the shell field [2]. These processes produce a shell that makes up to about 90% of larval dry body weight [7]. Prodissoconch I stage larvae are also called “D-larvae”, as the larval shell has a distinct D-shaped form. After the initial shell has formed, the larvae become planktotrophic and feed on microalgae for 2–3 weeks before developing into pediveliger larvae. When competent, pediveliger larvae settle on hard substrates and metamorphose into sessile juvenile oysters. At this life stage, shell mineral composition changes from aragonite to the calcite isoform of calcium carbonate [3, 8].

During these major life stage transitions, large-scale changes occur in the cellular biochemistry of the calcifying tissue (e.g. [9–12]). It is likely that these physiological and biochemical changes are linked to transcriptomic transitions, where for example transcripts coding for shell matrix proteins, ion pumps and other processes are up-regulated for shell formation, while others could be simultaneously down-regulated. Li et al. [13] showed that differential gene expression in the larvae of the pearl oyster, *Pinctada fucata*, occurs mostly during transitions between early developmental stages, such as from trochophore to D-shaped larva. Zhang et al. [10] reported that a fibronectin-like transcript and chitin synthase were highly expressed at the initiation of shell formation in larval *C. gigas*. While the bone-morphogenetic-protein (BMP) [14] signalling pathway has been hypothesized as an initiator of these changes [15–17], a number of studies have also been conducted on individual candidate genes putatively directly involved in biomineralization in oysters; for example tyrosinases have been suggested as having a function in periostracum formation and biogenesis [18, 19] and cyclases have been suggested to control intracellular calcium and bicarbonate ion concentrations [20, 21].

As several recent studies have shown a disconnect between gene expression and protein expression [22, 23], quantification of protein content provides an important link between gene expression changes and cellular physiology. In oysters, Huan et al. [9] compared proteins in non-calcifying trochophore larvae to calcifying D-shaped larvae and found 50 differentially expressed proteins, which they divided into the four categories “cytoskeletal components”, “biochemical regulators”, “cell proliferators” and “protein modification factors”.

On the United States (US) west coast, high mortalities of Pacific oyster larvae have recently occurred in conjunction with upwelling of deep water that is under-saturated in aragonite, causing a significant loss of income for oyster hatcheries and farmers [24–26]. Carbon dioxide (CO_2_)-acidified seawater can cause shell deformations and reductions in shell size of developing larvae (e.g. [27–29]) and delays in the initiation of shell formation (e.g. [27, 30–32]). The delay in shell formation of oyster larvae exposed to CO_2_-acidified seawater is affected by seawater aragonite saturation state (Ω_ARAG_) [5, 33, 34] and/or possibly by the ratio of bicarbonate to hydrogen ions at sites of calcification [34, 35].

Concurrent with shell formation, larval oysters are undergoing a complex transition from trochophore to veliger larvae, a process which invokes a myriad of physiological and transcriptomic changes [10]. In this study, we have capitalized on the delay in initial shell development of oyster larvae under acidified seawater conditions in order to identify genes that are correlated with shell calcification during this early developmental phase. We have compared gene expression profiles during early shell development of larvae in ambient (Ω_ARAG_ ≈ 2.5–3.0) seawater with those of genetically similar larvae in acidified (Ω_ARAG_ ≈ 1.0–1.25) seawater during the first 18 h post-fertilization in order to identify expression of genes previously known to be involved in shell formation, but also expression of novel putative genes coding for shell matrix proteins, as well as other processes associated with shell formation. This is the first study to use a high temporally resolved sampling scheme (every two hours) while assessing global gene expression changes due to Ω_ARAG_ stress in *C. gigas* larvae, an investigation which could provide a better understanding of how oyster populations may respond to environmental change. Additionally, this study could provide insight into potential targets of natural selection under future ocean acidification scenarios.

## Results

### Water chemistry

In replicate experiment 1, the partial pressure of carbon doixide (*p*CO_2_) in ambient conditions ranged between 462.8–731.0 μatm (μ = 564.9, s.d. = 65.6), while in the treatment it ranged between 1325.8–1724.5 μatm (μ = 1515.4, s.d. = 104.1) (Table 1; Additional file 1: Table S1). Aragonite saturation state ranged between 2.08–2.78 (μ = 2.53, s.d = 0.18) in ambient cultures, while in the treatment replicates it ranged between 1.06–1.31 (μ = 1.19, s.d. = 0.07) (Table 1). One sample from the ambient group (6 h post-fertilization, replicate A) was found to contain low Ω_ARAG_ water, potentially as the result of a mistake during filling of the culturing vessel, and was thus treated as a low Ω_ARAG_ treatment sample for the gene expression analyses. The exclusion of certain cultures resulting in lack of replication within certain time points is not of major concern from a statistical perspective due to our choice of regression analysis across all time points.

**Table 1:** Mean (±SD) temperature, salinity, total alkalinity (peq kg^− 1^), total CO_2_ (TCO_2_), partial pressure CO_2_ (*p*CO_2_), bicarbonate (pmol kg^− 1^), carbonate (pmol kg^− 1^), pH (pH_T_ = pH on the total scale) and saturation state of aragonite (Ω_ARAG_) for control and high *p*CO2 seawater treatments across two experiments rearing *C.gigas* larvae from 2 to 18 h post fertilization

		Parameter								
		Temp. (°C)	Salinity (ppt)	Alkalinity (μeq kg^−1^)	TCO_2_ (μmol kg^− 1^)	*p*CO_2_ (μatm)	HCO_3_^−^ (μmol kg^− 1^)	CO_3_^2−^ (μmol kg^− 1^)	pH_T_	Ω_ARAG_
Experiment 1	Control	25.8 ± 0.49	30.4 ± 0	2259 ± 11	2053 ± 13	565 ± 65.6	1882 ± 21	154.7 ± 11.4	7.92 ± 0.04	2.53 ± 0.18
	High *p*CO_2_	25.9 ± 0.28	30.4 ± 0	2285 ± 14	2225 ± 11	1515 ± 104.1	2109 ± 10	72.6 ± 4.5	7.54 ± 0.03	1.19 ± 0.07
Experiment 2	Control	26.1 ± 0.23	30.4 ± 0.04	2251 ± 18	2003 ± 11	449 ± 30.8	1808 ± 15	182.4 ± 10.6	8.01 ± 0.03	2.99 ± 0.17
	High *p*CO_2_	26.3 ± 0.28	30.4 ± 0.12	2284 ± 14	2194 ± 13	1242 ± 109.2	2071 ± 14	87.5 ± 6.8	7.62 ± 0.04	1.44 ± 0.11

In replicate experiment 2, the *p*CO_2_ in ambient conditions ranged between 405.0–521.6 μatm (μ = 449.0, s.d. = 30.8), while in the treatment it ranged between 1103.0–1397.9 μatm (μ = 1241.9, s.d. = 109.2) (Table 1). Aragonite saturation state ranged between 2.63–3.25 (μ = 3.00, s.d. = 0.175) for ambient conditions while in the treatment it ranged between 1.27–1.60 (μ = 1.44, s.d. = 0.115) (Table 1). In this experiment one sample from the low Ω_ARAG_ group (6 h post fertilization, replicate A) was found to have an abnormally low Ω_ARAG_ (0.688). As the variance in all other replicates was very low, we interpret this as a post-experimental sterilization failure of the water sample before analysis and not a true treatment effect. Carbonate chemistry in static culture systems is strongly influenced by biologic metabolism within the culture unit(s) and, as such, inherent differences in stocking rate (biomass) result in variability of seawater carbonate chemistry. Despite notable variation in seawater *p*CO_2_ between replicates for our experiments, Ω_ARAG_ levels were consistently and substantially different between treatments: > 2 for ‘ambient’ conditions and ≈1.0–1.5 for ‘treated’ seawater. No trend of decreasing Ω_ARAG_ with time was observed (Table 1, Additional file 1: Table S1).

### Shell deposition

**Fig. 1:**
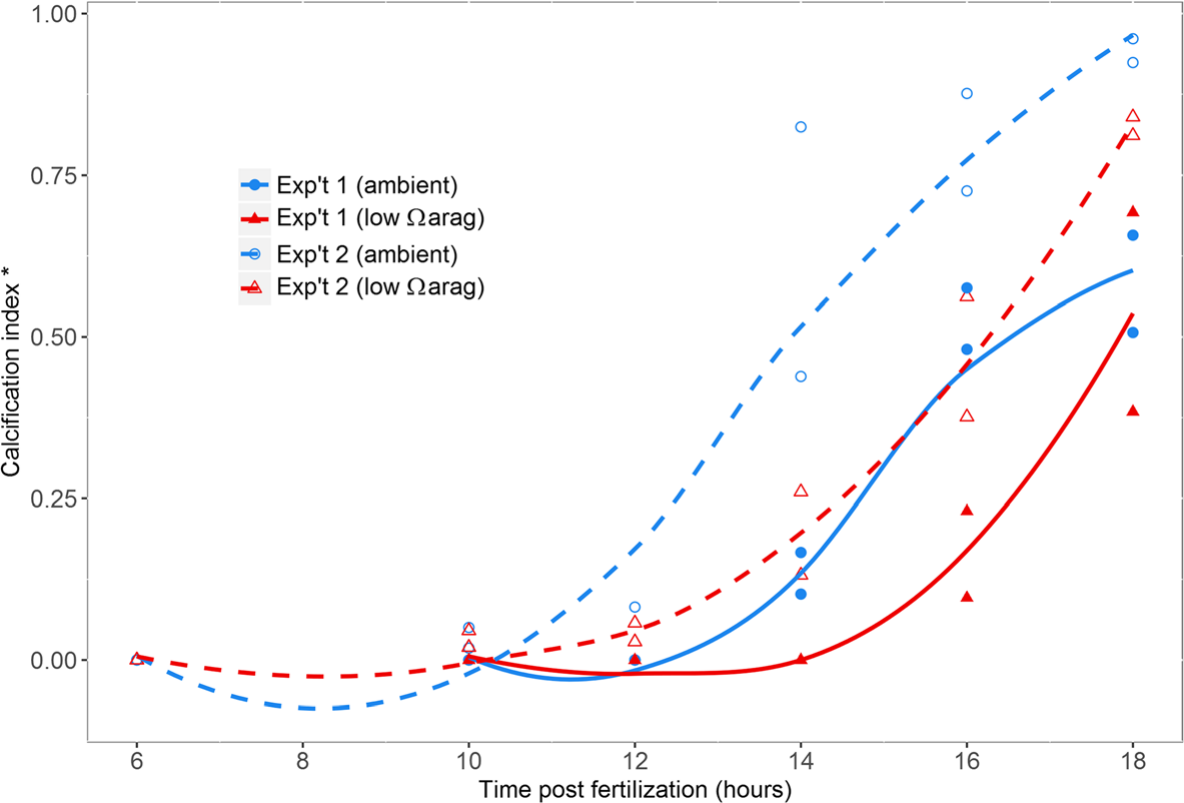
Calcification index of larval cultures from 6 to 18 h post fertilization. Calcification index (CI) is calculated as: CI = (FC + (PC ∗ 0.5))/TL, where FC, PC and TL denote the numbers of observed fully calcified, partially calcified and total larvae from each sample, respectively. Ambient (control) and low Ω_ARAG_ (treatment) conditions are represented in blue and orange respectively, with locally estimated average (LOESS) trends represented by lines. Experiment 1 is displayed as solid lines and filled points, experiment 2 is represented by dashed lines and unfilled points. Symbols of the same type within each time point correspond to the two independent replicate cultures as specified in the “Methods” section

Both timing of onset and rate of shell calcification was significantly different between seawater treatments and experiments (*p* < 0.05, Additional file 2: Table S2). In replicate experiment 1, larvae in ambient conditions began to form aragonite crystals at 14 h post-fertilization (μ_(CI)_ = 0.13, s.d. = 0.05) (3% fully calcified, 20% partially calcified), while larvae in low Ω_ARAG_ treatments did not start calcifying until 16 h post fertilization and then in much lower proportions than in ambient conditions (Fig. 1). At 18 h post fertilization, the proportion of calcified larvae in the low Ω_ARAG_ treatment reached similar levels to that of larvae in ambient conditions μ_(CI)_ = 0.58 ± 0.11 and 0.54 ± 0.22 for ambient and low Ω_ARAG_ treatments, respectively (61% part- or fully calcified vs 62% in ambient).

In replicate experiment 2, the same general calcification pattern was seen as in experiment 1 (Fig. 1), although larvae in both ambient conditions and low Ω_ARAG_ treatment began to partially calcify to a small extent as early as 10–12 h post fertilization (≈7% partially calcified in each). At 14 h, however, only larvae in the ambient treatment were fully calcified (48%) and at 16 h 74% were fully calcified in the ambient while only 22% were fully calcified in the low Ω_ARAG_ treatment. Similar to the results of replicate experiment 1, this difference diminished at 18 h (97% part- or fully calcified in ambient conditions vs 88% in the low Ω_ARAG_ treatment).

### Bioinformatic analyses of RNA transcripts

Samples were sequenced with 50 bp single-end reads, ranging from 13.2–77.4 Mreads sample^− 1^ (μ = 39.0 Mreads). After quality trimming and removal of residual adapter sequences, a mean of 97.3% of the reads were retained, with a mean quality score of 36.1 and mean length of 47.3 bases, considering data from both replicate experiments (Additional file 3: Table S3). Due to low output of the first sequencing run for replicate experiment 1, most of the samples were sequenced one more time, more than doubling the number of reads in this replicate experiment, with the exception of one 10 h replicate of the ambient treatment and both 10 h replicates of the low Ω_ARAG_ treatment, as well as both 12 h replicates of the ambient and one 12 h replicate of the low Ω_ARAG_ treatments, which were not re-sequenced. Unfortunately one of the ambient treatment replicates at 16 h in experiment 1 was lost during the library preparation protocol.

The fraction of reads that mapped uniquely to one position in the genome coding regions ranged from 3.1% to 38.0% (μ = 25.7%). This rather low fraction was likely due to the method of extraction of RNA in bulk from seawater, which would also extract RNA from a variety of micro-organisms. The fraction of duplicate reads ranged from 6.48% to 24.79% (μ = 18.72%). After removal of duplicate reads, sequencing depth ranged from 9.50–57.3 Mreads/sample (μ = 30.5 Mreads) (Additional file 4: Figure S1).

### Differentially expressed transcripts

**Fig. 2:**
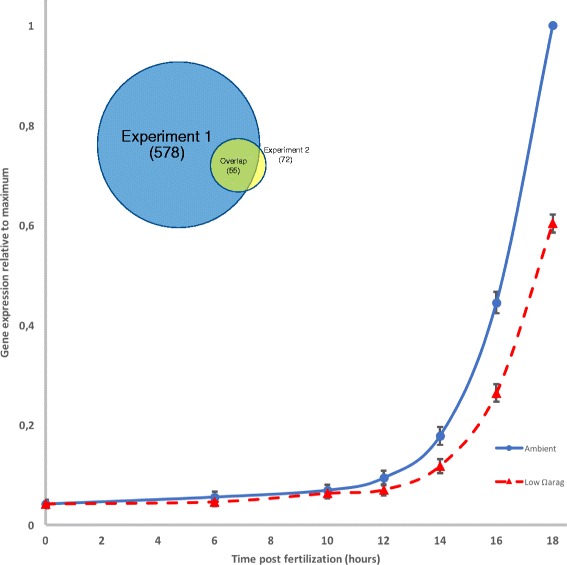
Summary of replicate experiment 1 expression in transcripts with significant time*treatment effect shared by both replicate experiments (Mean ± SEM, *n* = 55). Individual transcript expression is given in Additional file 5: Figure S2. Expression in ambient water is given in the solid line, low Ω_ARAG_ in the dashed line. Expression levels have been normalized by maximum expression level (1) for each transcript. Inset Venn diagram shows the number of significant transcripts shared among the replicate experiments

Filtering out transcripts with low expression values, low variances and ones not showing a positive expression * time interaction term in a generalized linear model (*p* < 0.05), left 5448 transcripts in experiment 1 and 4030 transcripts in experiment 2. From these datasets, 578 transcripts showed a significant (time ∗ treatment) interaction effect in the log linear model: log(y) = β_0_ + β_1_time + β_2_treatment + β_3_(time ∗ treatment) in experiment 1 after a Benjamini-Hochberg false discovery rate correction (FDR = 0.05%), while there were 72 transcripts in experiment 2 (p < 0.05). Fifty-five of the transcripts were shared between the two experiments (Inset in Fig. 2; Additional file 5: Figure S2), all of which show higher expression levels in the ambient than in the low Ω_ARAG_ treatment (Fig. 2). Out of these, 31 had an InterPro annotation [36] through the genome sequence, and 25 had a Gene Ontology (GO) functional annotation associated with them. In total, 37 were attached to some form of annotation, and all of these could be classified into one of four categories: Metabolic genes (*n* = 3), Transmembrane Proteins (transporters) (*n* = 8), Shell Matrix Proteins (*n* = 16) and Protease Inhibitors (*n* = 10) (Table 2). Overrepresented GO categories (within the “Molecular Function” category) in this list are: “endopeptidase inhibitor/regulator activity” (GO:0004866/GO:0061135; corrected *p*-value 3.35*10^− 10^), “serine-type endopeptidase inhibitor activity” (GO:0004867; corrected p-value 1.68*10^− 10^), “serine-type endopeptidase activity” (GO:0004252; corrected p-value 0.0251) and “serine-type peptidase/ hydrolase activity” (GO:0008236/GO:0017171; corrected p-value 0.0223) (Additional file 6: Figure S3). Looking closer at the transcripts within each of these categories, they code for the extracellular metalloprotease matrix protein “Papilin” (CGI_10,020,818 / CGI_10,021,289), as well as a variety of different protease inhibitors such as for example Antistasin (CGI_10021371), Trypsin inhibitors (CGI_10,015,381 / CGI_10,012,273 / CGI_10,020,625) and Cystatins (CGI_10013713 / CGI_10013715 / CGI_10013717).

**Table 2:** Thirthy-seven annotated transcripts with significant time * treatment effects in both replicate experiments, divided into four main functional categories. All are more highly expressed in ambient conditions

Metabolic transcripts	Annotation
CGI 10022578	Cytochrome P450
CGI 10025516	Sulfotransferase
CGI 10011094	Lipase
Transmembrane proteins	Annotation
CGI 10005173	Prominin
CGI 10024903	Transmembrane ion-channel
CGI_10009289	DEATH-like
CGI 10012122	Ganglioside activator protein
CGI 10007940	Caveolin
CGI 10011750	Transmembrane protein of unknown function
CGI 10012368	Pedal peptide
CGI 10022868	Leucine-rich glioma-inactivated protein
Shell matrix proteins	Annotation
CGI 10016584	C-type lectin
CGI 10024633	Chitin-binding protein
CGI 10024602	Fibrinogen, alpha/beta/gamma chain
CGI 10000698	Carbonic anhydrase / Nacrein-like protein
CGI_10027654	C-type lectin
CGI_10027048	Low-density lipoprotein receptor -like
CGI_10001361	EGF-like calcium-binding protein
CGI 10005422	Beta-lactamase-type transpeptidase
CGI_10016583	C-type lectin
CGI_10022862	Toll-like receptor
CGI_10024126	Thrombospondin
CGI_10025037	Calcium-binding EF-hand
CGI_10020619	EF-HAND 2
CGI_10013619	Temptin
CGI_10010907	Galactose-binding protein
CGI_10007447	Collagen alpha-6(VI) chain-like
Protease inhibitors	Annotation
CGI 10016790	Metalloproteinase inhibitor I3 5
CGI 10015381	Peptidase S1A, chymotrypsin-type
CGI 10018666	Cystatin-A2
CGI 10012273	Peptidase S1A, chymotrypsin-type
CGI 10010153	Protease inhibitor, Kazal-type
CGI 10020625	Peptidase S1A, chymotrypsin-type
CGI 10010888	Protease inhibitor, Kazal-type
CGI 10025096	Proteinase inhibitor I14/I15, hirudin/antistatin
CGI 10020818	Proteinase inhibitor I2, Kunitz metazoa
CGI 10021289	Proteinase inhibitor I2, Kunitz metazoa

### Weighted gene correlation network analysis

**Fig. 3:**
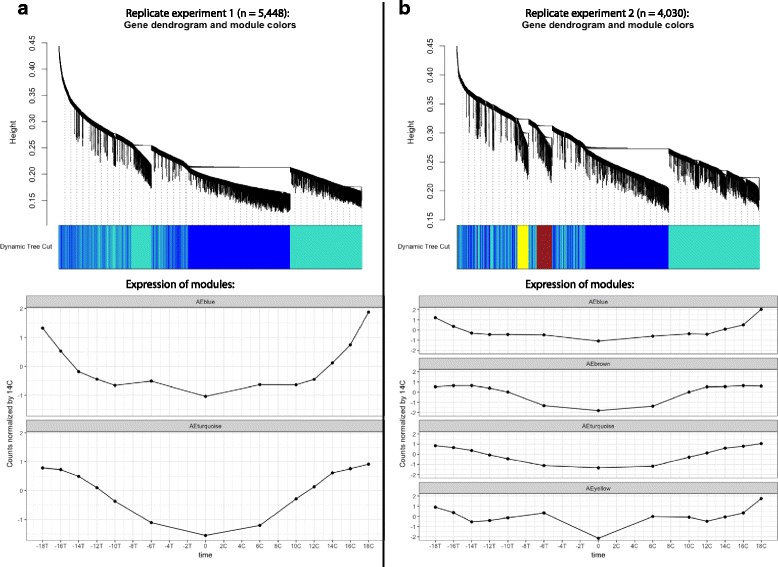
Weighted gene correlation network modules and normalized expression levels. Input data have been pre-filtered to include only transcripts showing a positive time-coefficient, a variance > 1 and total counts > 10. Correlation dendrograms are on top, expression of modules below. Expression plots list time-point zero in the middle, expression in ambient (Control: C) to the right and expression in low Ω_ARAG_ (Treatment: T) to the left. (**a**) Replicate experiment 1, dendrogram on top shows that transcripts are grouped in two modules, of which the “blue” module has higher expression in ambient after 14 h post-fertilization; (**b**) Replicate experiment 2, dendrogram on top shows that transcripts are grouped in four modules, of which the “blue” module has higher expression in ambient after 14 h post-fertilization

Clustering the expression data from the 5448 transcripts from replicate experiment 1, after filtering out transcripts that were found not to be positively correlated with time and with low variance, rendered two major clusters, of which one showed temporal differences in expression patterns between ambient conditions and low Ω_ARAG_ treatment (“blue” in Fig. 3a), whereas the other one did not (“turquoise” in Fig. 3a). Focusing on the “blue” cluster, it contained 2592 transcripts, with the list of genes being significantly enriched (Gene-score resampling multiple-test corrected *p* < 0.05) for 47 different GO categories (10 Biological Processes, 9 Cellular Components and 28 Molecular Functions) (Additional file 6: Figure S3). In replicate experiment 2, the transcripts clustered into 4 co-expression clusters, one of which showed different expression pattern in the low Ω_ARAG_ treatment compared to ambient (“blue” in Fig. 3b). This cluster contained 1658 transcripts, and was enriched for 12 GO categories (1 Biological Process, 4 Cellular Components and 7 Molecular Functions), all of which were also enriched in replicate experiment 1 except for tubulin-tyrosine ligase activity (GO:0004835) and serine-type peptidase activity / serine hydrolase activity (GO:0008236 / GO:0017171) (Additional file 6: Figure S3). The lower number of significant categories reflect the decrease in statistical power due to lower sequencing depth in replicate experiment 2. These categories also include the ones enriched in the dataset of transcripts showing a significant (time ∗ treatment) interaction described above (Additional file 6: Figure S3).

The transcripts responsible for the enriched functional categories in the “blue” clusters generated by the WGCNA analysis, together with transcripts with significant (time ∗ treatment) interactions, can be classified into three different main functions: 1) Extracellular Matrix Formation (Dynein chains, Myosin, Tubulin, Tektin, Integrin, Fibrillin, Cadherin, as well as Chitin binding proteins), 2) Transmembrane Ion Transport and homeostasis (Potassium, Sodium, Calcium and Copper channels, “Atrial Natriuretic Peptide” and several Lyases (Adenylate and Guanylate cyclase)), and 3) Protease Inhibitors (Papilin, Tryptase, Cystatin, Antistasin, Metalloproteinase inhibitor 3, Carboxypeptidase inhibitor SmCl).

## Discussion

### Delay in shell deposition rates

Our results indicate that shell formation of *C. gigas* larvae is affected by OA conditions. Shell development rates were reduced at aragonite conditions of 1.06–1.31 and 1.27–1.60 in experiments 1 and 2, respectively. This finding is in agreement with reports by others who have shown that shell formation of Pacific oyster larvae is impacted at Ω_ARAG_ below 1.5 [33]. In both of our replicate experiments, the low Ω_ARAG_ treated larvae started forming their shells at a later time than in ambient conditions. There seems to be a particularly large difference at the 14 and 16 h time points, indicating a developmental delay for larvae exposed to low Ω_ARAG_ conditions. This is consistent with the results for larvae of bivalve mollusks (e.g. [27, 30, 37]) and of purple sea urchins [38], and suggests that the gene expression patterns correlated with shell formation have shifted as a result of exposure to low Ω_ARAG_ conditions.

### Differentially expressed transcripts

There are many more significantly differentially expressed transcripts between low Ω_ARAG_ and ambient treatments in the first replicate experiment than in the second. This is most likely a result of the fact that in the first replicate experiment sequencing depth is twice as high for most time points. Despite this difference, there remains a remarkable overlap between the two replicate experiments: 55 out of the 72 significantly differentially expressed transcripts from replicate experiment 2 are also significantly differentially expressed in replicate experiment 1. Interestingly, all of the annotated genes from this list can be divided into only four functional categories: Metabolic Functions, Transmembrane Proteins (transporters), Shell Matrix Proteins and Protease Inhibitors. The metabolic genes are too few to result in significant enrichments for any metabolic GO category, and are restricted to specific types of metabolism, especially lipid breakdown. This could be associated with faster calcification rates in ambient seawater, as is also shown by increased expression of ion transporters and matrix protein transcripts, or by a switch in energy allocation as reported in sea urchin larvae [22]. It is somewhat unexpected to observe such a high number of protease inhibitors in this list; however, this type of inhibitor plays a very important role in preventing proteins from being hydrolysed by endopeptidases, and could be involved in shell formation as a way of protecting shell matrix proteins as they are secreted to form the extracellular matrix for mineral deposition [39]. This would especially be the case for the metallo-proteinase inhibitors, such as papilin [40], which could potentially protect important protein – CaCO_3_ bonds. As aragonite formation is highly sensitive to the organisation of the shell protein matrix [41], degradation of some of the matrix proteins could cause the aragonite crystals to become deposited in a sub-optimal manner which would, in turn, affect the integrity of the shell. This could be a cause for the high numbers of deformed shells observed in low Ω_ARAG_ treatments (e.g. [27, 33]).

Several of the differentially expressed transcripts are from genes known to code for parts of the shell matrix, such as nacrein [42], papilin (also a metalloprotease inhibitor; [40]), chitin-binding protein [43] and a protein with a beta-lactamase domain that is known to be part of the shell matrix, but with a currently unknown function [44]. Nacrein has a carbonic anhydrase domain [42], and has previously been shown to be strongly expressed prior to the initiation of shell formation in blue mussels [45]. Furthermore, several calcium binding proteins are represented here, and quite a few proteins involved in extracellular matrix agglutination, such as lectin, collagen, EF-Hand, thrombospondin and fibrinogen.

Transmembrane proteins are also found in this list, such as the ion channel protein caveolin, that is known to be involved in subcellular compartmentalization and vacuolar organization [46] as well as prominin that is involved in the organization of plasma membranes [47]. Interestingly, this list also includes transmembrane proteins mostly known as neurotransmitters, such as a ganglioside activator protein, a leucine-rich glioma inactivated protein [48], the Aplysia “pedal peptide” gene [49] and a protein containing a DEATH-like domain [50]. These proteins could be involved in transport of ions across the plasma membrane, which in some cases can be associated with neuronal activity, but also in shell formation [51]. Altogether, this list paints an image of a machinery which binds calcium ions and synthesizes matrix proteins, then transports these components across the plasma membrane to the external environment while ensuring that the matrix proteins are not hydrolysed by proteases before calcium carbonate crystals have been deposited.

### Weighted gene correlation network analysis

The clustering approach allows us to “zoom out” from investigations of individual transcripts to instead get an overview of cellular functioning. The large number of transcripts falling into the co-expression cluster more highly expressed in the ambient than in the low Ω_ARAG_ treatment highlights that the cellular response to aragonite saturation stress involves many genes/pathways. While being more sensitive and thus causing more GO categories to be enriched, the clustering result agrees strongly with the result of the individual transcript analysis (significant time*treatment transcripts) in that the categories can be grouped into ion transport, protein synthesis, extracellular matrix proteins, and a few metabolic pathways (lipid breakdown). One type of transcript that is highly overrepresented in many of these categories is dynein (both cytoplasmic and axonemal). Dynein motor proteins are well-known major transporters of cellular components, and it is possible that these have a role in the transport of the building blocks of the shell matrix to the location where it should be deposited.

The GO category “viral capsid” is also enriched in replicate experiment 1. This could seem surprising at first sight, but these are all chitin-binding proteins and thus likely to be part of the shell formation machinery [43].

Transmembrane serine protease and antistasin are also commonly found in the enriched GO categories. These are genes known to have a part in immune defence and regulation of coagulation in other organisms [52, 53], and is possible that these genes also have a role in the control of the deposition of shell matrix proteins in larval oysters.

### Comparison with previous work

As part of the work describing the *C. gigas* genome, gene expression profiles of developing oyster larvae were produced [10]. Mining this resource (https://www.ncbi.nlm.nih.gov/bioproject/PRJNA146329) for expression levels in the 55 transcripts exhibiting significant time*treatment interactions in our data, we see consistent patterns with those of Zhang et al. [10] who found that all of these transcripts also started being expressed during the transition between “trochophore” and “early D shaped larva” stages, some of these to extremely high expression levels. Interestingly, however, Zhang et al. [10] found that some of these transcripts only reached their maximum expression levels at later developmental stages, hours or even days after the end of our experiment, while others were highly expressed only in D-larvae. It is reasonable to expect that there are different mechanisms involved in the initial process of shell nucleation, compared to later shell formation stages where aragonite layers are being deposited on pre-existing layers, thus it makes sense to pay special attention to genes expressed in only early D-larvae. Unfortunately, however, the transcripts which Zhang et al. [10] found to be most highly expressed at this stage are not annotated (e.g. CGI_10010907, CGI_10022681, CGI_10014978, CGI_10022681), thus their functions are not known. Further experiments using molecular biological tools to elucidate the functions of these genes could be an interesting subject of future research.

Using a proteomics approach, Huan et al. [9] identified a number of proteins differentially expressed between trochophore (11 h post-fertilization) and D-larvae (21 h post-fertilization). Some of the expressed proteins could also be categorized as matrix proteins (e.g. tubulin, tropomyosin) and protein modification factors. Differences between their results and those of our study could have been due to a number of factors, for example the timing of sampling points, differences in oyster population used, or a lack of correlation between transcript and protein expression [22].

In a recent article, Wang et al. [21] found that adenylyl cyclase was an important mediator of bicarbonate ion concentrations and intracellular pH in low Ω_ARAG_ conditions in adult *C. gigas*. Interestingly, it seems that the same mechanism is also important in larval oysters, as shown by both adenylyl and guanylyl cyclases being commonly found in our enriched GO categories. Guanylyl cyclase is also known to mediate the import of calcium ions into cells, through production of cyclic guanosine monophosphate (cGMP), which can act to keep cGMP-mediated calcium ion channels open [20]. Alkaline phosphatases, such as adenylyl cyclase, have also been found to be highly expressed in the gastropod *Biomphalaria glabrata* just prior to the onset of shell formation [54].

**Fig. 4:**
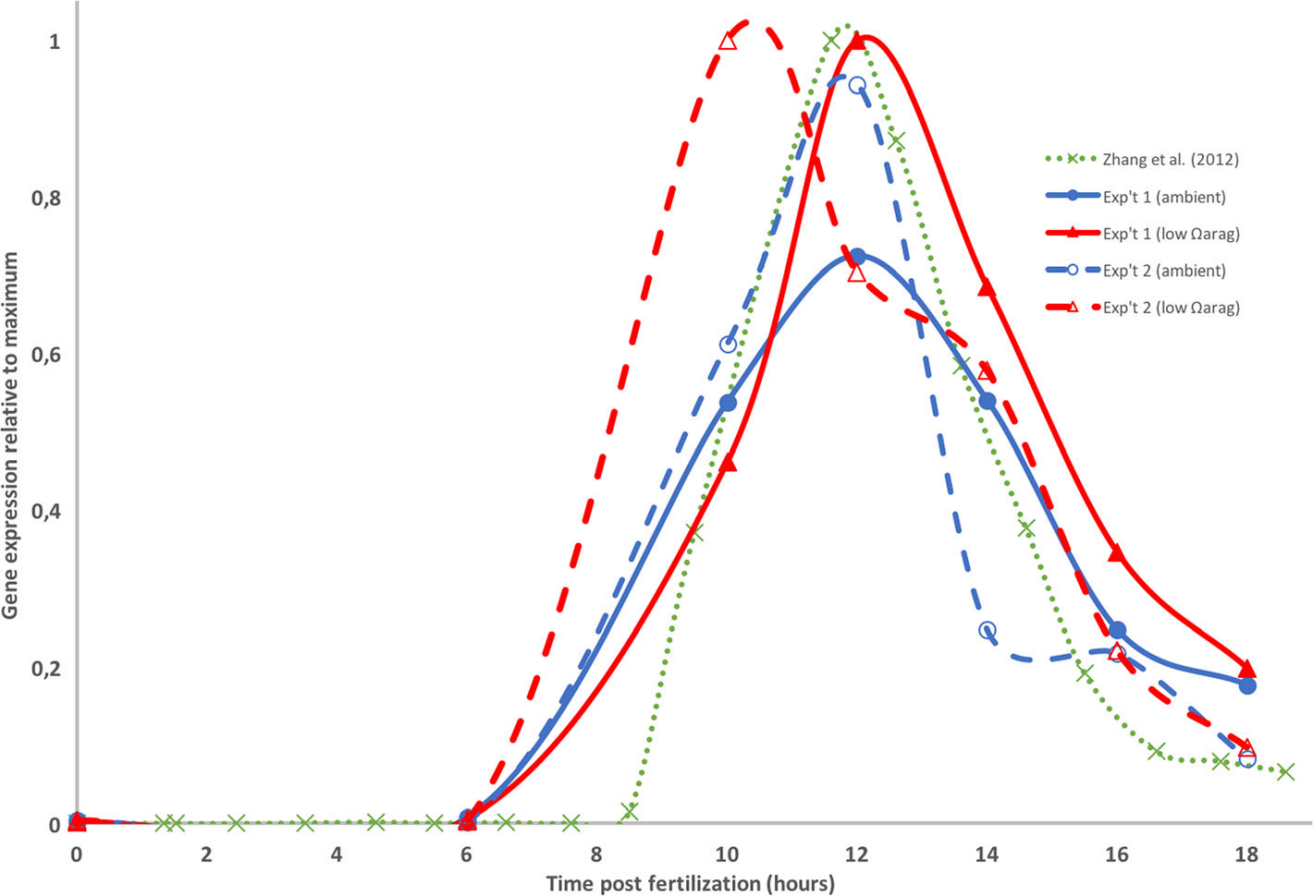
Tyrosinase (contig CGI_10007793) expression in our experiments (Experiment 1, solid line; Experiment 2, dashed line; Ambient in blue, low Ω_ARAG_ in red) vs Zhang et al. (2012) (Green dotted line). Expression values have been normalized by the maximum value for each experiment (1)

Huan et al. [19] identified a tyrosinase gene (cgi-tyr1) in *C. gigas* which was highly expressed in the trochophore and D-larvae stages but not later in larval development. More recently, Yang et al. [55], found that the homolog in *C. angulata* (Ca-tyrA1) was expressed especially in trochophore larvae, but was also significantly upregulated in a high *p*CO_2_ treatment (3000 ppm). The authors concluded that this was likely in response to high *p*CO_2_ induced shell damage, and that Ca-tyrA1 was involved in larval shell repair. Wang et al. [56] proposed the involvement of a tyrosinase gene (CGI_10017214) in the shell formation process following shell damage in adult *C. gigas*. An important role of tyrosinases in the first stages of shell repair in the blue mussel *Mytilus edulis*, has also recently been discussed [57]. Our data support previous work in that the tyrosinase gene (gene ID CGI_10007793; available at http://www.uniprot.org/uniprot/U5U0P0) is highly expressed before shell formation starts (10–14 h post-fertilization), perhaps in association with formation of the larval pellicle or periostracum [18, 55, 58], and then the expression level decreases rapidly. In addition, the transcripts are expressed more in the low Ω_ARAG_ treatment than in ambient during these hours and the larvae in low Ω_ARAG_ in replicate experiment 2 peak in tyrosinase expression several hours earlier than remaining treatments (Fig. 4). Examining the expression data from Zhang et al. [10] shows that there the tyrosinase contig (CGI_10007793) was highly expressed at 11.5–13.5 h (Fig. 4). In addition, this contig clusters (using the WGCNA approach as described above) in a co-expression cluster together with 11 other contigs, out of which several are cation channel proteins and potential matrix proteins (Table 3). These transcripts would be interesting candidates for further study of their role in the onset of shell formation.

**Table 3:** Contigs co-expressed with the Tyrosinase contig CGI_10007793

Contig ID	Annotation
CGI 10003647	EGF-like calcium-binding protein
CGI 10005120	NHL repeat containing protein-binding
CGI 10011377	Glucose/ribitol dehydrogenase
CGI 10012546	No annotation
CGI 10018671	Strictosidine synthase
CGI 10018819	Transient receptor potential cation channel
CGI 10018820	Similar to transient receptor potential cation channel
CGI 10022617	No annotation
CGI 10024194	Follistatin-like, protease inhibitor, Kazal-type
CGI_10028176	No annotation
CGI_10028233	Transient receptor potential cation channel

In search of regulatory proteins controlling larval shell formation, Liu et al. [17] found that the transcription factor GATA2/3 was expressed in the edge of the shell during trochophore and D-larval stages in *C. gigas*. In our dataset, GATA3 (GeneID: CGI_10013217) is most highly expressed at 6 h post fertilization, then decreases in expression with time, but there is no difference between levels under ambient and low Ω_ARAG_ conditions. Liu et al. [17] also concluded that the GATA genes might be involved in other functions rather than in shell formation, most notably they discussed the possibility of them being involved in haemocyte formation, as has been shown in scallops [59]. This could potentially be an interesting area of further research, as it has been shown that calcification is initiated inside haemocytes before the constituents are transported to the location of shell deposition [1]. Liu et al. [17] also proposed that GATA-3 could be part of the BMP signalling pathway, which has been implied as critical for shell formation in the gastropod *Lymnaea stagnalis* [60]. Other potential parts of the BMP pathway could consist of different types of growth factors. Liu et al. [16] investigated the expression patterns of the two transforming growth factors (TGF) cgi-smad1/5/8 and cgi-smad4, and found that the former was more highly expressed within the shell field than in other parts of the larvae during early shell formation, suggesting an involvement in shell formation through regulating transitions between different developmental stages in the early development of oyster larvae. In our data, cgi-smad1/5/8 (Gene ID: CGI_10014747) is highly expressed at all time points except in eggs, with no difference between ambient and treatment (Additional file 7: Figure S4), while cgi-smad4 (Gene ID: CGI_10000594) is highly expressed in eggs, then decreases in expression with time in replicate experiment 2, while in replicate experiment 1 it shows a spike in activity at 6 h post fertilization, after which expression levels decrease with time (Additional file 7: Figure S4).

## Conclusions

**Fig. 5:**
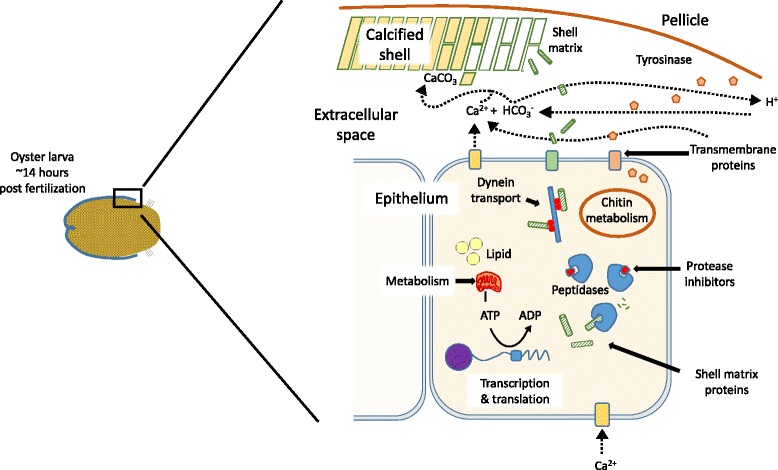
Schematic representation of biological and chemical processes at the leading edge of shell formation in a developing Pacific oyster larva at ~ 14 h post fertilization. Solid arrows indicate biological processes identified by differentially expressed transcripts, dashed arrows indicate transport of inorganic molecules into and out of the site of calcification

By examining differences in gene expression in oyster larvae during the period when shell formation is delayed by low Ω_ARAG_ (14–16 h post fertilization), together with previous findings of other studies, we can start obtaining a better understanding of the mechanism by which the prodissoconch I larval shell is initially deposited (Fig. 5). Although it is possible that this delay in shell formation is due to a general developmental delay, almost all of the differentially expressed transcripts observed in this study seem to be related to shell deposition. Our data suggest that the shell formation mechanism can be divided into three different main parts, namely 1) transport of ions across plasma membranes, 2) secretion of shell matrix proteins, and 3) production of protease inhibitors (Fig. 5). Increased expression of transcripts for calcium ion pumps suggests increased release of calcium ions into the epithelial space that would result in elevated aragonite saturation states. Dynein motor proteins may transport shell matrix proteins to this external space, facilitating and perhaps controlling the deposition rate and organization of shell aragonite crystals, although these observed expression changes could potentially also be due to other cellular processes. We also observed increased transcription of genes for chitin catabolism – chitin has been reported to be present in larval shells [43]. The role of the significant increase in transcripts for protease inhibitors in unknown, but one may hypothesize that they are important in preventing breakdown of proteins involved in shell formation (Fig. 5).

At later stages of development, it has been shown that metabolism is affected by aragonite saturation conditions, but this effect seems to be minor and associated with breakdown of lipid energy reserves during the period from 14 to 16 h post fertilization when larvae are starting the process of shell formation. Thus, production of shell matrix and other proteins would seem to be fueled by higher rates of lipid breakdown, based on increased transcription of genes coding for lipases.

Interestingly, we do not see any gene expression differences in genes previously hypothesized to be involved in the shell formation signalling pathway. This could be due to a lack of power due to low replication in this study, or it could actually be that the signalling pathway remains unaffected by the water chemistry, and that the detrimental effects of low Ω_ARAG_ on shell formation occurs downstream at the shell matrix level. We do, however, see a spike in expression of genes coding for tyrosinase prior to the onset of shell formation (10–12 h post fertilization), which is stronger in low Ω_ARAG_, as was recently also described in *C. angulata* [55]. This spike probably results in cross-linking of proteins and formation of the early larval pellicle. It is unlikely, however, that the pellicle can form a completely sealed space between itself and the outer shell-forming, epithelial layer of the developing larva by 14–16 h post fertilization. In support of this contention, Waldbusser et al. [7] reported that carbon isotopes of the early oyster larval shell were dominated by those from the external seawater medium and not from respired carbon. The lack of a sealed external epithelial space also suggests that hydrogen ions, formed by formation of carbonate, as well as calcium ions can also be exchanged by diffusion with the external seawater medium [7] (Fig. 5).

This study describes how low Ω_ARAG_ affects gene expression and shell formation of early oyster larvae. The identified genes, which show changes in expression as a result of OA conditions may play an important role in determining the capacity of oyster larvae to respond to future OA stress under natural and hatchery conditions.

## Methods

### Larval culture

Commercial broodstock of *Crassostrea gigas* was obtained from Netarts Bay, Oregon, USA, in August 2014. Recent publications have suggested that broodstock exposure to high *p*CO_2_ seawater may result, alternatively, in more robust larvae [61] or compromised ones [62]. In order to simplify our experiments, we chose to maintain all broodstock in ‘optimal’ conditions with pH ≈ 8.0–8.3 and Ω_ARAG_ > 2 (buffered with sodium carbonate) at a temperature of 19-20 °C prior to experimentation at the Hatfield Marine Sciences Center (HMSC), Oregon State University, Newport, Oregon, USA. Eggs from two females were collected through strip spawning and each sample was equally divided into four aliquots in seawater at ambient Ω_ARAG_ (> 2) and 25 °C. Each aliquot from each female was fertilized with sperm from one of four males. After one hour, fertilization was confirmed and excess sperm were removed by washing the eggs on a 20 μm mesh sieve, then equal numbers of fertilized eggs from each cross were combined. After pooling, the eggs were counted and ≈50,000 eggs were sampled for RNA by preserving them in RNAlater. Eggs were stocked in sealed 1 L glass jars approximately 2 h post fertilization at a density of 40 eggs ml^− 1^, as prior trials hatching *C. gigas* larvae up to 80 eggs ml^− 1^ had shown no adverse effects to density at this age. The larvae were reared in either ambient (Ω_ARAG_ ≈ 2.5–3.0) or low Ω_ARAG_ (Ω_ARAG_ ≈ 1.0–1.25) treatment conditions. The level of seawater acidification selected for the low Ω_ARAG_ treatment was chosen in order to increase the likelihood of generating sufficient larval developmental delay. In full salinity seawater (≈33 ppt), a *p*CO_2_ level of ≈1500 ppm results in Ω_ARAG_ of 1–1.5, a range that impedes shell formation [33] and is also routinely observed during periods of upwelling along the West Coast USA [25].

Seawater types for each treatment were prepared by filling two 200 L tanks with full strength seawater (≈32 ppt) ≈ 18 h before the start of each experiment and aerating them vigorously with outside (ambient) air to allow the seawater to equilibrate with ambient CO_2_ concentrations overnight. Next, a mixture of outside air and pure CO_2_ moderated by mass flow controllers (Alicat Scientific, Tuscon, AZ), was bubbled into the low Ω_ARAG_ seawater tank for ≈2 h until a pH of 7.5 was reached. Nominal pH values were measured using an Orion Star A211 pH meter with a Ross Ultra pH/ATC triode probe (Thermo Fisher scientific) calibrated with NBS buffers and standardized with a certified seawater reference (Batch 22, A.G. Dickson, Scripps Institution of Oceanography, US.). Seawater for both ambient and low Ω_ARAG_ conditions were transferred from corresponding storage tanks to sealed 1 L glass jars (VWR scientific, part no. 89094–014) filled with 800 mL seawater immediately prior to stocking with eggs and were sealed with a screw top lid. Cultures were treated prophylactically with 2 ppm of the antibiotic chloramphenicol to reduce bacterial respiration. Previous experiments had indicated that this antibiotic treatment had no effect on larval growth and survival [63]. Larvae were sampled at 6, 10, 12, 14, 16 and 18 h post fertilization from two independent replicate glass jars for both ambient and low Ω_ARAG_ conditions which results in a total of 24 samples.

At each sampling point, temperature, salinity and pH of the culture medium were measured, then sample water volumes from each duplicate vessel per treatment were siphoned through a screen (attached to the end of a silicone tube immersed in the culture vessel to prevent larval removal) into a 350 ml amber glass bottle. Each sample was preserved by the addition of 30 μl saturated HgCl_2_ and sealed with a polyurethane-lined metal crimp cap, until subsequent carbonate chemistry analysis was performed (see below). The remaining culture volume was sieved onto a 25 μm mesh screen to retain larvae, which were then re-suspended in 25 ml of ambient or low Ω_ARAG_ seawater, depending on their exposure treatment. Larval concentrations were estimated by aliquoting 30 μl (*n* = 3) volumes of vigorously agitated suspensions and counting larvae in the samples under a microscope.

At all sampling points, except at 6 h post fertilization, two to three hundred developing larvae were transferred to 20 ml shell vials with 10 ml of seawater, to which 250 μl of buffered formalin (pH 8.2) was added, for later analyses of shell deposition by means of cross-polarized light (CPL) microscopy (see below). Remaining larvae were concentrated by centrifugation (2 min at 4500 rpm) after addition of 10 ml distilled water to reduce larval buoyancy to facilitate pellet formation, after which all supernatant seawater was immediately removed. Larvae (ca. 10,000/sample) were then flash frozen in liquid nitrogen in order to break larval shells to allow RNAlater to quickly infuse into larval tissues. The time period from addition of freshwater to flash freezing of samples was less than 5 min to avoid effects of sampling on gene expression. Samples were stored in RNAlater and divided between two replicate 1.5 ml Eppendorf tubes for later transcriptomic analyses (see below). All tubes were maintained at 4 °C for 24 h, then stored at − 20 °C until RNA extraction.

The entire experiment was repeated a few days later using the same methodology but different broodstock (from the same source) was used and sperm from five males instead of four were added to batches of eggs from each of two females. In addition, eggs were stocked at a density of 20 eggs/ml (rather than 40 in experiment 1), resulting in ca. 5000 larvae per replicate culture sampled for transcriptomic analyses.

### Water chemistry analyses

Preserved water samples were analysed by the lab of Dr. Burke Hales at Oregon State University following the procedure outlined by Hales et al. [64] and Bandstra et al. [65] to obtain values for sample total dissolved carbon dioxide (TCO_2_), *p*CO_2_, and seawater pH, from which Ω_ARAG_ values were calculated. This method has been shown to be highly accurate, providing TCO_2_ and *p*CO_2_ estimates with < 0.2% and < 5% uncertainty, respectively [7].

### Shell deposition measurements

Larvae from each time point were analysed for calcification using cross-polarized light (CPL) microscopy as described by Waldbusser et al. [5]. Patterns of refractive light viewed under CPL indicated deposition of crystalline calcium carbonate (aragonite) in the process of shell formation. A fully formed shell is curved and the secondary refraction of the polarized light creates a “Maltese cross” pattern: a dark cross area over the center of the otherwise illuminated larval shell. In this way, we were able to classify larvae into three categories: non-calcified, partially calcified and fully calcified (shelled) larvae for each sample. From these data, a “calcification index” (CI) was calculated as: CI = (FC + (PC ∗ 0.5))/TL, where FC, PC and TL denote the numbers of observed fully calcified, partially calcified and total larvae from each sample, respectively (Fig. 1). In order to determine the statistical significance of difference in timing of calcification onset and rate of shell formation, calcification index data from hour 10–18 (period over which calcification occurred) was arcsine transformed and fit with a generalized linear model (GLM) with the formula: CI~β_Time_ ∗ β_Trt_ ∗ β_Exp_, where β_Time_, β_Trt_ and β_Exp_ represent estimates for time (hours post fertilization), treatment (seawater *p*CO_2_) and experiment number, respectively. The “full” model (with all interactions) was subsequently submitted to backwards stepwise AIC selection (“stepAIC” in R) to generate a final optimal fit model with the formula CI~β_Time_ + β_Trt_ + β_Exp_ + (β_Time_ x β_Trt_) + (β_Time_ x β_Exp_). Associated *p*-values for each parameter were calculated on Type I sum of squares based on a chi-squared distribution.

### Transcriptomic analyses

RNAlater-fixed larvae were transported to the Sven Lovén Centre for Marine Sciences, Tjärnö, Sweden, where total RNA was extracted using a Qiagen RNeasy kit (Qiagen, Hilden, Germany), following the standard protocol. RNA concentrations were measured using a QuBit 2.0 RNA fluorometric assay (Thermo Fisher Scientific, Waltham, MA, USA) and integrity was assessed with a 3-(N-morpholino)propanesulfonic acid (MOPS) denaturing agarose gel. Complementary DNA (cDNA) libraries were prepared using the Illumina TruSeq v2 mRNA sample prep kit (Illumina, San Diego, CA, USA), following a standard protocol. Briefly, mRNA was isolated with poly-A selection, followed by cDNA synthesis, Illumina standard index adapter ligation and a brief PCR reaction. Concentrations of the cDNA libraries were measured using a QuBit DNA High-sensitivity assay (Thermo Fisher Scientific, Waltham, MA, USA) and fragment length distributions were assessed using an Agilent TapeStation with a D1000 tape (Agilent, Santa Clara, CA, USA). cDNA libraries were multiplexed by equimolar pooling (6 or 7 samples/pool), and were then sent to the Swedish National Genomics Infrastructure’s SNP & SEQ platform in Uppsala for Illumina HiSeq 2500 sequencing (8 lanes; 50 bp Single-End sequencing; Illumina, San Diego, CA, USA).

Bioinformatic analyses were performed on the University of Gothenburg computer cluster Albiorix (http://albiorix.bioenv.gu.se) where raw reads were trimmed of low-quality (Q < 20) ends and Illumina adapter sequences were removed using the fastx toolkit (http:// hannonlab.cshl.edu/fastx_toolkit/). All reads were mapped to the oyster genome v9 coding regions (available at “ftp.ensemblgenomes.org/pub/metazoa/release-34/fasta/crassostrea_gigas/cds/Crassostrea_gigas.GCA_000297895.1.31.cds.all.fa.gz”) using the Burrows-Wheeler Aligner (bwa) (http://bio-bwa.sourceforge.net/ [66]), allowing for four mismatches, after which count data for each contig was extracted using a custom script (all scripts available at https://github.com/DeWitP/Bioinformatic_Pipelines/tree/master/RNA-Seq_materials/scripts). Only reads mapping uniquely to one genome contig were considered. Duplicates were initially removed from alignment files in order to assess the effects of duplicate reads. After determining that the proportion of duplicates was low and correlated to sequencing depth, we decided to keep the duplicate reads, as is customary. To be able to compare count data across samples with differences in sequencing depth, the count data were scale-normalized using the estimateSizeFactors function in the DESeq package [67] in R.

Scale-normalized count data were then analysed in R independently for each replicate experiment (*n* = 2) in several different ways. Firstly, transcripts with low variance (< 1) and with low counts (total counts < 10) were filtered out, after which exponential curves were fitted to the count data, keeping only transcripts showing increased expression with time in the ambient treatment. This was done in order to filter out transcripts with peak expression levels in the newly fertilized eggs or prior to the onset of shell formation, as well as transcripts showing no variation in expression over the experimental period.

With the filtered datasets, we then fitted log-linear curves of the type: log(y) = β_0_ + β_1_time + β_2_treatment + β_3_(time ∗ treatment) to the transcripts of larvae from the ambient and low Ω_ARAG_ treatments, while forcing expression to be 0 at time point 0 (time of fertilization) (i.e. β_0_ = 0), and keeping transcripts with a significant (*p* < 0.05) time*treatment interaction after a Benjamini-Hochberg false-discovery rate correction (Fig. 2). The lists of significant transcripts were annotated when possible using the oyster genome annotation and compared across replicate experiments for overlap (Additional file 5: Figure S2).

Transcripts with significant effects of time*treatment in both replicated experiments were examined for non-random distributions of function using a functional enrichment Gene Score Resampling (GSR) analysis in ErmineJ [68]. Briefly, GSR analysis examines the distribution of functions (given by the Gene Ontology (GO) annotation from Zhang et al. [10]) in the transcripts of interest, and compares that to what would be expected from a random draw of all the gene models in the genome. Significant deviations from the expected random distribution can then be interpreted as evidence for a biological importance of function with regard to experimental treatments. The advantage of this approach is that it can identify patterns of biological relevance in lists of transcripts, while overcoming the issue of noise at transcript-level in gene expression data.

The filtered datasets were also analysed for gene regulatory co-expression clusters, using a weighted gene correlation network analysis (WGCNA package in R [69]) with Pearson correlation scores. This type of analysis is useful in that it summarizes all of the variation in a large dataset into correlation clusters, assigns transcripts to the different clusters and allows for visual examination for clusters showing expression patterns of interest. In order to do this, counts were first normalized across transcripts by dividing by the mean count level at 14 h post fertilization in the ambient treatment (point chosen arbitrarily as mean counts were never 0). Transcripts belonging to clusters showing different expression profiles between ambient and low Ω_ARAG_ treatments were extracted and analysed for GO enrichment in ErmineJ as described above.

## Additional files

Additional file 1: Table S1. Aragonite saturation states in ambient and low aragonite saturation state treatments in the two replicate experiments. (PDF 516 kb)

Additional file 2: Table S2. Results of Type I sum of squares for GLM modelling the response variable Calcification Index (CI) as a function of the independent variables β_time_, β_Trt_, and β_Exp_ representing time (hours post fertilization), seawater treatment and experiment number, respectively, along with two 2-way interactions between time and treatment (β_time_ x β_Trt_) and time and experiment (β_time_ x β_Exp_). All parameters are deemed significant on a *p* = 0.05 threshold. (DOCX 13 kb)

Additional file 3: Table S3. Quality statistics for all samples. Sample names include: Time post-fertilization (in hours), treatment (C = ambient, T = low Ωarag), and Replicate number (A or B). (XLSX 12 kb)

Additional file 4: Figure S1. Mapping statistics for all samples, with and without duplicate reads. Sample names include: Time post-fertilization (in hours), treatment (C = ambient, T = low Ωarag), and Replicate number (A or B). (XLSX 17 kb)

Additional file 5: Figure S2. Expression of transcripts with significant time x treatment effect in both replicate experiments. (PDF 1564 kb)

Additional file 6: Figure S3. Summary of functional enrichment results of the WGCNA clusters showing differences in expression across treatments (“blue” clusters in Fig. 3), as well as the 55 transcripts showing significant time*treatment effects (Fig. 2). (XLSX 12 kb)

Additional file 7: Figure S4. Relative (to maximum) expression of (A) cgi-smad1/5/8 and (B) cgi-smad4 in the two replicate experiments. (PDF 576 kb)

## Abbreviations

AIC: Akaike Information Criterion
BMP: bone-morphogenetic-protein
bwa: Burrows-Wheeler aligner
CaCO_3_: Calcium carbonate
cDNA: complementary DNA
cGMP: cyclic guanosine monophosphate
CI: Calcification index
CO_2_: Carbon dioxide
CPL: Cross-polarized light
FDR: False discovery rate
GLM: Generalized linear model
GO: Gene ontology
GSR: Gene score resampling
HMSC: Hatfield Marine Sciences Center
MOPS: 3-(N-morpholino)propanesulfonic acid
OA: Ocean Acidification
*p*CO_2_: partial pressure of carbon dioxide in seawater
TCO_2_: total dissolved carbon dioxide
TGF: transforming growth factor
US: United States of America
WGCNA: Weighted Gene Correlation Network Analysis
Ω_ARAG_: Aragonite saturation state
