# Supplementary material for: Gene expression correlated with delay in shell formation in larval Pacific oysters (Crassostrea gigas) exposed to experimental ocean acidification provides insights into shell formation mechanisms
Source: BMC Genomics. 2018 Feb 22;19:160. doi: 10.1186/s12864-018-4519-y (PMC5824581; doi:10.1186/s12864-018-4519-y)
Supplement: Supplementary file 1 — Aragonite saturation states in ambient and low aragonite saturation state treatments in the two replicate experiments. (PDF 516 kb) [file 12864_2018_4519_MOESM1_ESM.pdf]

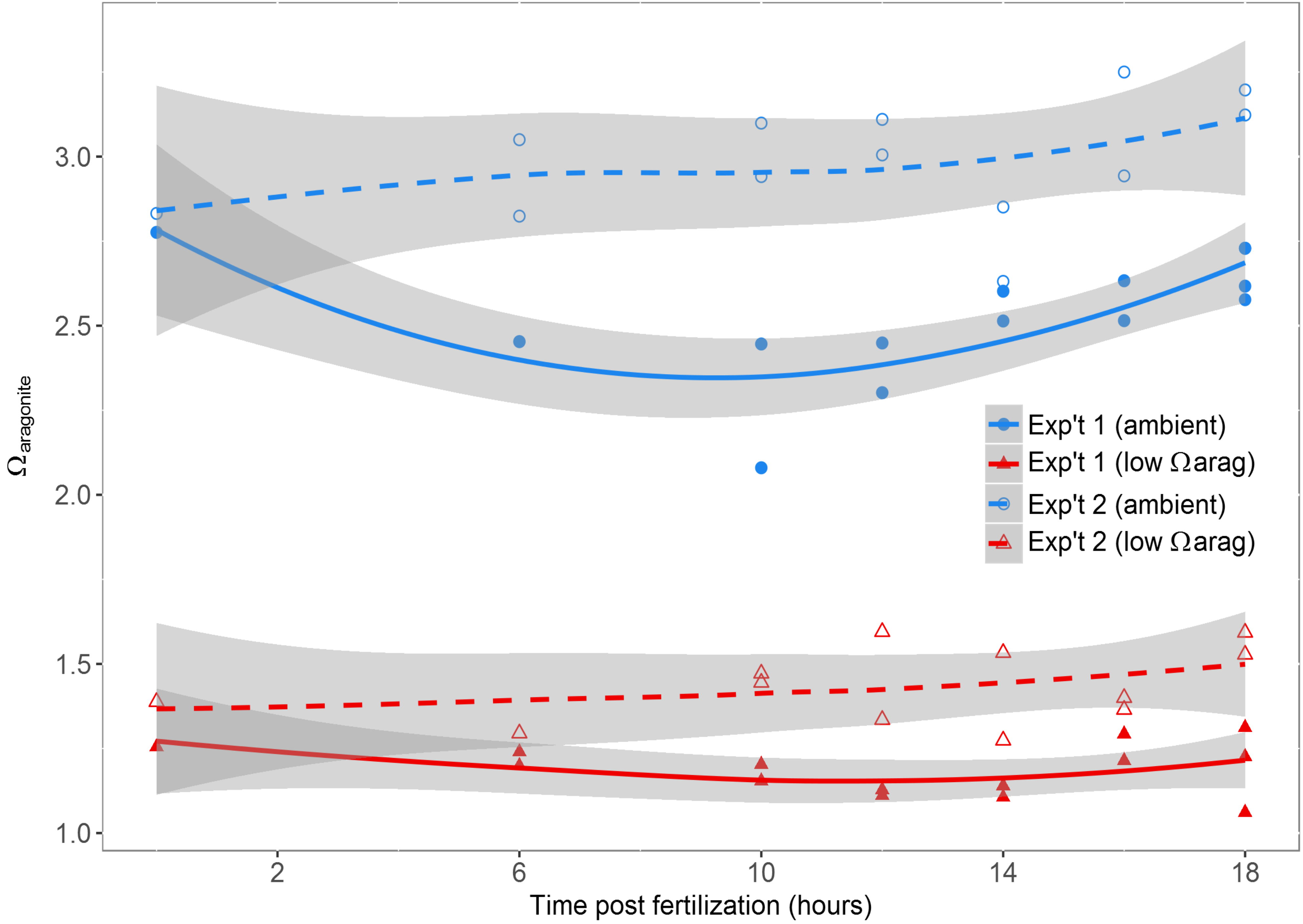

Additional file 1: Figure S2. Aragonite saturation states in ambient and low aragonite saturation state treatments in the two replicate experiments.
