# Supplementary material for: Gene expression correlated with delay in shell formation in larval Pacific oysters (Crassostrea gigas) exposed to experimental ocean acidification provides insights into shell formation mechanisms
Source: BMC Genomics. 2018 Feb 22;19:160. doi: 10.1186/s12864-018-4519-y (PMC5824581; doi:10.1186/s12864-018-4519-y)
Supplement: Supplementary file 2 — Results of Type I sum of squares for GLM modelling the response variable Calcification Index (CI) as a function of the independent variables βtime, βTrt, and βExp representing time (hours post fertilization), seawater treatment and experiment number, respectively, along with two 2-way interactions between time and treatment (βtime x βTrt) and time and experiment (βtime x βExp). All parameters are deemed significant on a p = 0.05 threshold. (DOCX 13 kb) [file 12864_2018_4519_MOESM2_ESM.docx]

| Formula: $CI\sim{}_{Time}+{}_{Trt}+{}_{Exp}+(_{Time} x {}_{Trt})+(_{Time} x {}_{Exp})$ | | | | | |
| --- | --- | --- | --- | --- | --- |
| Source | Df | Deviance Residuals | Df | Residual Devience | P_r_(>Chi) |
| NULL |  |  | 39 | 6.1996 | - |
| Time | 1 | 3.834 | 38 | 2.3656 | < 2.2e-16 |
| Trt | 1 | 0.3235 | 37 | 2.0421 | 0.0005781 |
| Exp | 1 | 0.696 | 36 | 1.3461 | 4.46E-07 |
| Time:Trt | 1 | 0.1056 | 35 | 1.2405 | 0.0492618 |
| Time:Exp | 1 | 0.3119 | 34 | 0.9286 | 0.0007264 |

**Additional file 2: Table S1**. Results of Type I sum of squares for GLM modelling the response variable Calcification Index (CI) as a function of the independent variables *β_time_*, *β_Trt_*, and *β_Exp_* representing time (hours post fertilization), seawater treatment and experiment number, respectively, along with two 2-way interactions between time and treatment (*β_time_* x *β_Trt_*) and time and experiment (*β_time_* x *β_Exp_*). All parameters are deemed significant on a p=0.05 threshold.
