# Supplementary material for: Gene expression correlated with delay in shell formation in larval Pacific oysters (Crassostrea gigas) exposed to experimental ocean acidification provides insights into shell formation mechanisms
Source: BMC Genomics. 2018 Feb 22;19:160. doi: 10.1186/s12864-018-4519-y (PMC5824581; doi:10.1186/s12864-018-4519-y)
Supplement: Supplementary file 5 — Expression of transcripts with significant time x treatment effect in both replicate experiments. (PDF 1564 kb) [file 12864_2018_4519_MOESM5_ESM.pdf]

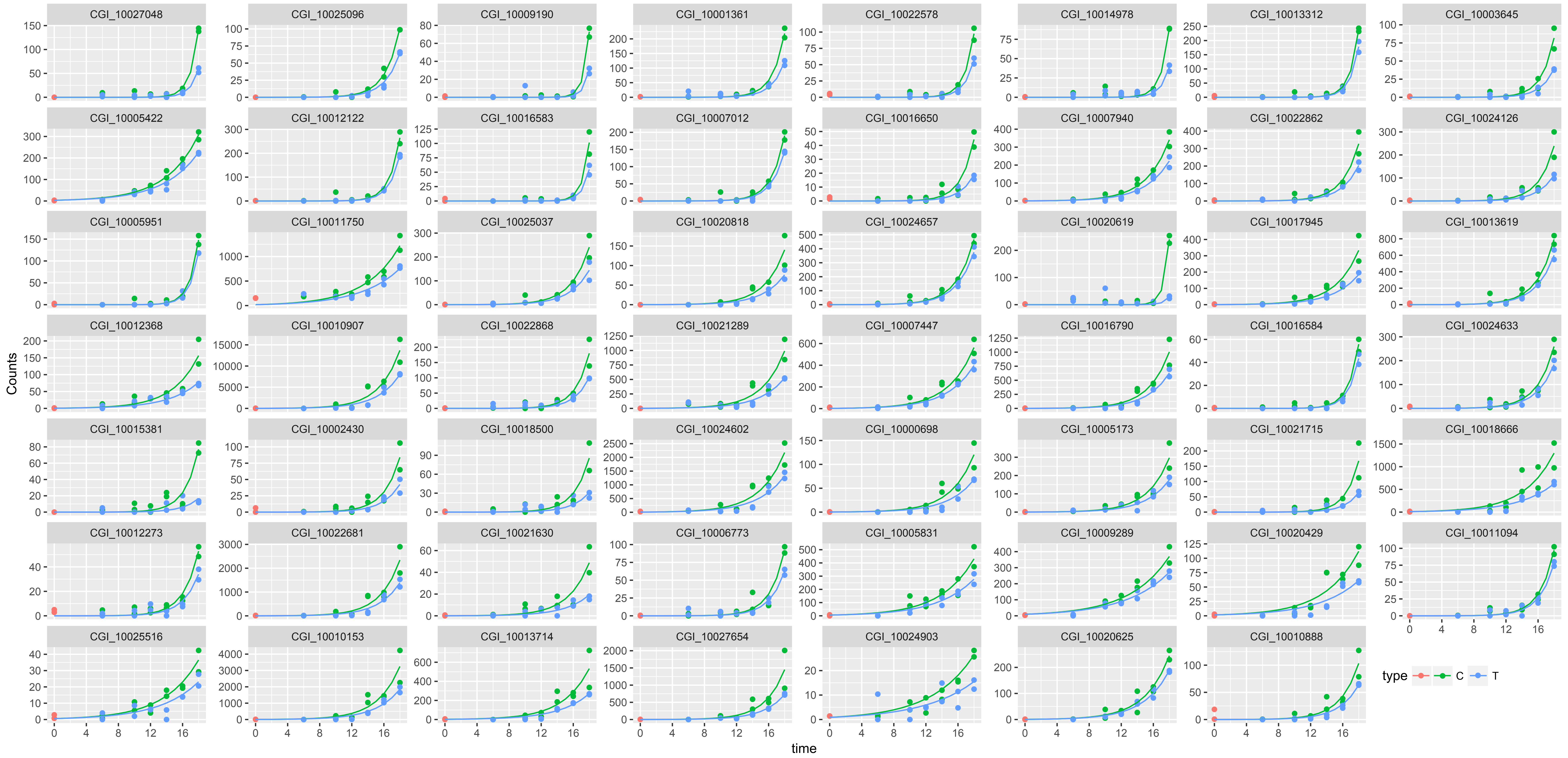

Additional file 5: Figure S1. Expression of transcripts with significant time x treatment effect in both replicate experiments.
