# Supplementary material for: Gene expression correlated with delay in shell formation in larval Pacific oysters (Crassostrea gigas) exposed to experimental ocean acidification provides insights into shell formation mechanisms
Source: BMC Genomics. 2018 Feb 22;19:160. doi: 10.1186/s12864-018-4519-y (PMC5824581; doi:10.1186/s12864-018-4519-y)
Supplement: Supplementary file 7 — Relative (to maximum) expression of (A) cgi-smad1/5/8 and (B) cgi-smad4 in the two replicate experiments. (PDF 576 kb) [file 12864_2018_4519_MOESM7_ESM.pdf]

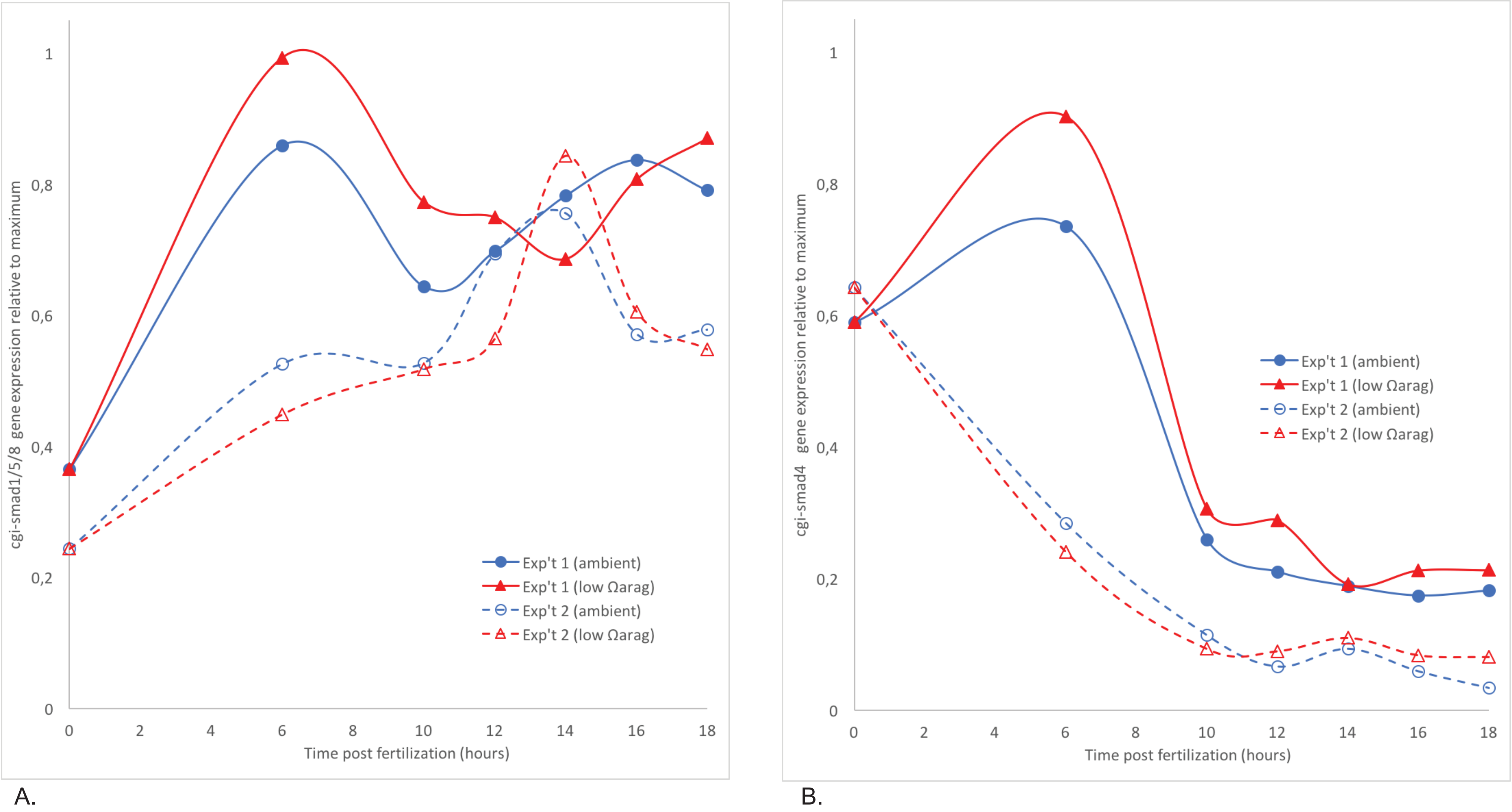

Additional file 7: Figure S3. Relative (to maximum) expression of (A) cgi-smad1/5/8 and (B) cgi-smad4 in the two replicate experiments.
